# Experiences in applying skills learned in a mental health first aid training course: a qualitative study of participants' stories

**DOI:** 10.1186/1471-244X-5-43

**Published:** 2005-11-09

**Authors:** Anthony F Jorm, Betty A Kitchener, Stephen K Mugford

**Affiliations:** 1 ORYGEN Research Centre, University of Melbourne, Locked Bag 10, Parkville, Victoria 3052, Australia; 2 Centre for Mental Health Research, Australian National University, Canberra, ACT 0200, Australia; 3 Qualitative and Quantitative Social Research, Gungahlin, ACT 2912, Australia

## Abstract

**Background:**

Given the high prevalence of mental disorders and the comparatively low rate of professional help-seeking, it is useful for members of the public to have some skills in how to assist people developing mental disorders. A Mental Health First Aid course has been developed to provide these skills. Two randomized controlled trials of this course have shown positive effects on participants' knowledge, attitudes and behavior. However, these trials have provided limited data on participants' subsequent experiences in providing first aid. To remedy this, a study was carried out gathering stories from participants in one of the trials, 19–21 months post-training.

**Methods:**

Former course participants were contacted and sent a questionnaire either by post or via the internet. Responses were received from 94 out of the 131 trainees who were contacted. The questionnaire asked about whether the participant had experienced a post-training situation where someone appeared to have a mental health problem and, if so, asked questions about that experience.

**Results:**

Post-training experiences were reported by 78% of respondents. Five key points emerged from the qualitative data: (1) the majority of respondents had had some direct experience of a situation where mental health issues were salient and the course enabled them to take steps that led to better effects than otherwise might have been the case; (2) positive effects were experienced in terms of increased empathy and confidence, as well as being better able to handle crises; (3) the positive effects were experienced by a wide range of people with varied expectations and needs; (4) there was no evidence of people over-reaching themselves because of over-confidence and (5) those who attended were able to identify quite specific benefits and many thought the course not only very useful, but were keen to see it repeated and extended.

**Conclusion:**

The qualitative data confirm that most members of the public who receive Mental Health First Aid training subsequently provide support to people with mental health problems and that this support generally has positive effects.

## Background

Community surveys in a range of countries show a high prevalence of mental disorders, but with many cases not receiving appropriate professional help [1]. As well as this unmet need with clinical disorders, there is the problem of how to help the large number of people with sub-clinical symptoms. These people are a source of considerable disability in the population and have a high risk of progressing on to clinical disorders [2]. The problem of unmet need is not uniform within the community. Some sub-groups, such as people living in rural areas, have even lower access to services [3,4].

In order to help overcome the problems of high prevalence, unmet need for treatment, the impact of sub-clinical symptoms, and the uneven geographic distribution of services, there is a need to consider solutions that lie outside formal health services. One approach is to widen the base of people with some knowledge and skills in helping people with mental health problems. Examples of such an approach, which have either been implemented or proposed, include the use of lay people as counsellors in telephone help-lines [5], web sites giving mental health information or therapy directly to the public [5], training of community members, including parents, in how to assist people in crisis situations [6,7], and training of human service providers in rural areas in how to support people suffering from mental disorders [8].

An approach of this sort, which has recently been developed in Australia and has spread to a number of other countries, is Mental Health First Aid (MHFA) training [9,10]. The philosophy behind this approach is that people with mental health problems can potentially be assisted by those in their social network. There is evidence that better social support reduces risk of developing mental disorders and improves outcomes for people experiencing disorders [11,12]. However, potential helpers often lack the confidence and skills to provide basic help. Supporting this view, a recent national survey of the Australian public found that many people had a less than adequate range of first aid responses to people with mental disorders [13]. The MHFA course follows the model that has been successfully applied with first aid for physical disorders. It trains members of the public to give early help to people with developing mental health problems and to give assistance in mental health crisis situations.

Two controlled trials have been carried out to evaluate the MHFA course. The first of these involved 301 employees of government departments who were randomized to either receive the training immediately or to be wait-listed for 5 months before undertaking the training [14]. The trained group improved more than the wait-list control group in terms of confidence in providing help to others, likelihood of advising people to seek professional help, concordance with health professionals about treatments, stigmatizing attitudes, and mental health of the participants themselves.

The second controlled trial was carried out with 753 members of the public in a large rural area of south-east Australia [15]. In this trial, the catchment of an area health service was divided into 16 local government areas. Eight of these areas received the course immediately and the other eight were controls and placed on a waiting list to receive the training later in the year. Relative to controls, people who did the course showed better recognition of disorders from case descriptions, decreased social distance towards people with mental disorders, became more like health professionals in their beliefs about what treatments are likely to be helpful, had greater confidence in providing help to someone, and were more likely to actually provide help.

While these controlled trials showed benefits of the training, the quantitative measures did not seem to fully capture the effects. There have been many instances where participants have told stories, sometimes dramatic ones, of how they used their skills following the course to help someone. It may be more natural for participants to report their experiences in the form of stories, because that is the way they are organized in memory [16], than to answer more abstract questions about them. Furthermore, the quantitative measures did not capture the effects on the recipients of first aid, whether positive or negative, because it was not possible to identify and survey these recipients. By analyzing the stories it is possible to get some indirect information about the effects on the recipients. We therefore sought to gather such stories in a systematic way using participants from the second controlled trial and to analyze the major themes that emerged.

## Methods

### Sample

The sample was drawn from 131 participants in a cluster randomized trial (ISRCTN53887541) [15]. These were the participants who were in the intervention group (N = 416) and were known to have completed every session of the 9-hour training course in March-April 2003. Those excluded either did not attend the course after random assignment (n = 76), only attended some sessions (n = 33) or had missing attendance data (n = 176). The participants were phoned by one of two research assistants who asked them to complete an additional questionnaire. Out of the 131 approached, 94 actually completed the questionnaire. The questionnaires were completed in November 2004–January 2005, which was 19–21 months after finishing the training course.

### Questionnaire

A survey questionnaire was developed to achieve the following: (1) Gather data on respondents with regard to age, gender and education; (2) Ascertain whether the respondent had, or had not, experienced a post-course situation where someone seemed to have a mental health problem; (3) For those who *had *experienced such a situation, a variety of questions were asked which allowed the respondent to describe that experience; and (4) For those who *had no*t had such an experience, the questionnaire asked them a few questions about expectation and about confidence with such a situation if it were to arise in the future.

For those who *had *experienced a post-course situation where someone seemed to have a mental health problem, the questions ran as follows: Could you tell us something about the situation(s) and the problems you believed the person(s) was experiencing? Were you able to do anything specific to help the person (s) you believed was suffering the mental health problem(s)? [For those who could not help] What was the reason(s) that you were not able to help that person(s)? Can you give us any examples of something you did? What do you think were the effects on that person/s of what you did? Can you give us any examples of how your relations with that person/s, or your feelings towards them, have changed? Do you think this change had any effect on the person/s, either good or bad? How (if at all) has doing the MHFA course changed how you relate to or feel about the person(s) suffering from that mental health problem?

For those who *had not *experienced a post-course situation where someone seemed to have a mental health problem, the questions ran as follows: Is this what you would have expected or is it somewhat surprising not to have come across such a situation? If you were now to come across someone who you believed was suffering from a mental health problem, how well prepared would you feel to deal with that? How has doing the MHFA course changed how you relate to or feel about a person(s) suffering from mental health problems? Is there anything else you would like to say about the MHFA course and its value for you?

### Survey procedure

Participants were given the option of completing the questionnaire either on-line or on paper. If the former, the participant was asked to provide an email address and was subsequently emailed the URL which gave the questionnaire at a *WebSurveyor*™ site. By clicking on the URL, the respondent was connected to an on-line facility. This site allows built in switching and skipping, so that only the questions applicable to a given respondent appear on the screen. S/he either selected a button based response for closed-ended questions or typed in free text for open-ended responses. The data generated were downloaded as Excel spreadsheets. Participants who chose to do a paper questionnaire had it mailed out. The paper responses were entered into Excel and the two data sources combined into one. Participants who failed to complete a questionnaire were given a reminder either by email or post, as appropriate.

### Analysis

To give independence in the evaluation from the developers of the Mental Health First Aid course, the company *Qualitative and Quantitative Social Research *was contracted to develop the questionnaire and carry out the analysis. The analysis consisted of three parts: (1) description of the respondents (2) quantitative analysis of frequencies of response and cross-tabulations of respondent characteristics with types of response, and (3) qualitative analysis identifying themes behind responses to each question and selecting stories that illustrate the range of experiences reported. The raw material of the stories was examined in print form. First, the material was read through by one of the authors (SKM) and broad themes were developed. Each major element of an answer was then tagged with a code to identify the themes and the material sorted to ensure that any themes that were identified in the analysis and any quotes that were to be used to illustrate these themes were genuinely representative of the material. Particular emphasis was laid upon ensuring that those themes that emerged frequently were highlighted in the reported analysis. As far as possible, stories quoted are in the exact words of the respondents – some very minimal editing has been undertaken to improve the flow of the story and to tidy up grammar, syntax and spelling.

## Results

### Characteristics of the respondents

There were 94 respondents to the questionnaire, with 56 responding by mailing a paper copy and 38 completing it on-line. The respondents were predominantly female (75/94) and had an age range from 21–74 years (mean 51 years). With regard to education, 31 had a university degree. Looking at previous experience with mental illness, 29 reported a personal experience, 21 a family experience, 8 reported both, and 36 neither. Compared to the full sample participating in the intervention group of the trial [15], these respondents tended to be better educated (33% vs 21% with a degree), but were similar in age and gender.

### Quantitative findings

Table 1 shows the distribution of responses to the basic questions asked. Most respondents had experienced, post-course, a situation where someone seemed to have a mental health problem. Of the 73 who had experienced a mental health related situation, 79% said they had definitely been able to help, 17% said they thought they had been helpful and only 7% were unsure. Of those who had not clearly had any encounter with a mental health issue post course, most felt they could cope well or moderately well with such a situation if it arose.

**Table 1:** Frequencies of responses to questions

**Question**	**Response**	**N with Response**
Experienced a post-course situation involving mental health problem	Yes	73
	No, Not Sure, No Response	21
	Total	94
If had an experience: able to help?	Yes, definitely	44
	Yes, I think I was helpful	10
	Unsure	4
	No	0
	No response	15
	Total	73
If not had an experience: surprised?	Yes	2
	No	9
	Don't know	7
	No response	3
	Total	21
If not had an experience: feel prepared now?	Cope well	9
	Cope moderately well	12
	Unsure or don't know	1
	Total	22

The data were also examined to see if there were any apparent patterns of association, either between independent variables, such as age, sex, education or form of response (on paper vs. on line) or between independent and dependent variables (qualities of the experience). The principal method used was to compile contingency tables and then examine various measures of association – as well as visual inspection – as a guide. Formal chi-square tests were not used because cluster effects in the sample violated the assumption of independent observations. Associations are not reported here, because only one association of any apparent size emerged: an association between levels of reported education and how the survey was completed, with on-line completion being associated with higher levels of education. However, the lack of other associations shows that the MHFA course was equally valuable to a wide range of people, irrespective of age, gender and education.

### Qualitative findings

Answers to each of the open-ended questions could be analysed as a block of data in their own right. However, it was also possible to connect the responses from consecutive questions to create short stories that illustrate the value of the course.

### Stories about first aid

Table 2 presents three stories that are focused mainly around problems that took place within the intimate social network of friends and family, while Table 3 presents three that examine issues that arose in the work setting (broadly defined).

**Table 2:** Stories of providing first aid that involve the intimate network

**Story 1**
*The situation: *[I was] living with a girl with manic depression. *What you did: *I recommended some professional help *Effects on that person: *She is now on medication and getting on well with work (etc) again. *How relations changed: *We no longer live together, she has moved away from where her problems essentially evolved. I now see her in a different light: she was once a fun-loving person, now she rarely leaves home. *Longer term effects on the person: *She says now that she is much happier than before. *How the course has changed you: *With the MHFA course and my social science degree, I understand the way people act and behave and why they do so. *Anything else: *It gave me a basic understanding and some valuable, simple information to help me get hold of the concepts of mental health.

**Story 2**

*The situation: *My daughter was suffering from drugs and stress and had a psychotic episode. *What you did: *I went to counselling with her and to see the psychiatrist with her. In the end she wanted to be on her own with nothing to do with my husband or me and we "backed off". I did keep in contact via fax: it was one sided but made it easier to keep the dialogue alive. *Effects on that person: *My daughter made contact again of her own accord and I believe it was because I never broke contact with her. *How relations changed: *I don't think we will ever have the same close relationship as before but we do talk on the phone and see each other at least once a month. I have tried and continue to try not to be too protective, which is what she felt I was. *Longer term effects on the person: *I think my daughter has realised that we love her, she is no longer on drugs and I believe that she no longer has the stress she had before. *How the course has changed you: *Doing the MHFA made me realise that we were not alone and that there were lots of other people in the same boat. So I did not feel so guilty and it helped my husband and me as well as my daughter knowing we were going to the course. *Anything else:* I think it is, no, I know it is an excellent course that has helped so many people and I hope MHFA course will always continue.

**Story 3**

*The situation: *[The man concerned was experiencing] severe depression and anxiety due to marriage/family break-up and child custody problems. *What you did: *I persuaded him to seek counselling. I tried to listen and advise and I also gave essential financial assistance. *Effects on that person: *He did seek counselling and also presented at a mental health unit as an in-patient for a few days but has since discontinued regular counselling. *How relations changed: *I now have a little more understanding but I feel there is still an underlying serious problem and I find it difficult to know how to best handle potentially explosive situations. *Longer term effects on the person: *There was a temporary effect for the good. He may also be more willing to seek help in the future if needed now he has experienced what the mental health unit can offer. *How the course has changed you: *I am somewhat more understanding and make more allowance for irrational behaviour, etc, but it is still not always easy. *Anything else: *I am very pleased I did the course and it has made me aware not only of the problems people have due to mental health, but of the help that is available if only the person will seek it.

**Table 3:** Stories of providing first aid that involve the work setting.

**Story 4**
*The situation: *[I'm currently] working with a family who lost a child due to an accident. 'Mum' has had a history of post natal depression and is now experiencing an episode of depression that has lasted in excess of six months. She is taking medication but sometimes cannot afford to get it. *What you did: *I offered non-discriminatory, non-judgmental support. I made gentle suggestions about ways she might get child to school, helped her get a Christmas tree up (for the kids) which alleviated her guilt around not feeling like having a Christmas and so on. *Effects on that person: *I think she was very grateful that someone seemed to understand and was willing to help her achieve little things rather than expect her to "get her act together." I have become someone who she doesn't have to put on an act for or pretend she isn't home. *How relations changed: *Previously when people tried to 'help' she would not answer the phone or pretend she wasn't there. This has not completely changed: recently I visited at an agreed time and no-one answered the door. I could hear music and wondered whether she was in fact there but didn't open the door. *Longer term effects on the person: *I feel that we have developed a relationship that is based on gaining small positive outcomes. She is a long, long way from where she would like to be, but we now have a dialogue based on her being able to tell someone how she is feeling and having someone listen. *How the course has changed you: *It has made me aware of the signs and symptoms of depression. I have been able to work from where the client is at rather than having specific expectations of their contribution to assistance. *Anything else: *I believe that the MHFA course has been particularly valuable to me. I am not a trained counsellor or therapist but in my work with families whose children have disabilities it is likely that some of my families will suffer from depression from time to time.

**Story 5**

*The situation: *I work for a Job Network Provider. Many of the long term unemployed suffer from depression and other mental health problems. One example a client was always injuring himself on jobs and on the latest job (the employer said) he wouldn't stop crying. There was also another female client who was suffering from Crohn's Disease who had always been very nervous and worried. *What you did: *I referred the woman with Crohn's disease to a holistic GP (as her previous GP did nothing). This new GP referred her to counselling, got her on anti depressants and is looking after her health. *Effects on that person: *I saw the difference in this person and she felt the difference of feeling better and learning to cope with life stressors. Last time I saw her she had improved a lot. *How relations changed: *I have always been a naturally caring person. I care for that client and most of my clients. *Longer term effects on the person: *Good! She not only was getting better but realised she can make a difference to her own life by using the appropriate skills and getting help. She also realised that there are people who care and she was not alone. *How the course has changed you: *It confirmed my previous knowledge of Mental Health and gave me a few more ideas of what to do when someone needs help. *Anything else: *I think it should run regularly as the population needs to learn how to deal with these sorts of situations and it creates an awareness and perhaps takes away the fear of the unknown.

**Story 6**

*The situation: *I was working for a charity organisation for work experience to achieve a Diploma of Community Welfare. I was an interviewer for people of low income or on benefits, when a client entered the room (I was not allowed to participate but just sat there and listen to what the other two interviewers said). This client spoke very fast and told us about the people who he lived with burning his clothes in the lounge room in the middle of the floor. The other two interviewers did not know how to react in this situation: I could not control myself I had to speak up. I just asked him a simple question as whether he was taking medication, he said he wasn't. Then the other two interviewers asked about his doctors, etc. *What you did: *By just asking a simple question about medication made the other interviewers realise he had a mental illness. *Effects on that person: *He told me that he didn't need his medication any more and he was no longer ill. However, I think that he must have gone to the doctor because I saw him a few weeks later in a different situation and he was acting quite normally. *How relations changed: *I really do not think they have changed as I only know him as a client and I have a good understanding towards people I interview. The only change would be the improvement because of medication. *Longer term effects on the person: *The change would be for the better because of medication. *How the course has changed you: *There has always been a stigma attached to mental illnesses, it has opened my eyes about how I and others feel toward these people. I am more understanding. *Anything else: *Most people do not have an understanding of mental illness. If more people could participate in the Mental Health First Aid course it would be a better world. I think the course was a great value to me personally.

In Table 2, Story 1 shows how the course helped someone cope with a challenge, reacting constructively to a partner with a mental disorder. The story is not one with a conventional happy ending – they did not in fact "live happily ever after" – but it does illustrate how the respondent was able to act constructively and make sense for himself about what happened.

Story 2 is somewhat similar in that it centres on dealing with a person with a mental disorder in an intimate, family relationship. Again there is a benefit with regard to increased understanding and self-knowledge. Here, however, we also see that it enabled the respondent to take actions that preserved a relationship that otherwise was in serious jeopardy.

Story 3 is slightly less intimate, but concerns the way a person responded to a friend who was experiencing mental health issues. The main benefits here are focused around the short term – it helped to get the man concerned to get counselling – and in palliating the emotional impact of the challenge. While the respondent does not see that having been at the course solved the problem, clearly he finds the short term and palliative effects valuable.

The three workplace stories (Table 3) are quite different in context, even though they share some themes: the first comes from a person who works directly in a helping profession, the second from someone who works in a context where clients have ancillary problems, while the last example comes from someone who was carrying out work experience.

In Story 4, the respondent clearly identifies benefits for him and for a client with whom he was working who experienced mental health issues. While he does not feel he fully solved anything, he is clearly able to show how deploying the skills he had acquired led to a positive set of developments and small wins.

In Story 5, a person who works with socially disadvantaged clients mentions that he sees many people with mental health issues. Choosing one recent case as an example, he shows how increased understanding and awareness led to taking positive steps that benefited the client.

Story 6 introduces a quite different aspect. Here we see that while the intervention the respondent took was positive for a client, and also increased the respondent's self-awareness and positive feelings, a major benefit is that the knowledge acquired on the course positively role modelled something for co-workers and helped them meet a challenge.

### Analysis of themes in open-ended questions

In this section, each open ended-question is presented and the results analysed and illustrated with examples.

#### Could you tell us something about the situation(s) and the problems you believed the person(s) was experiencing?

As the following illustrations show, some people took the course because of existing problems with friends and family in their life, others because voluntary work brought them into contact with people experiencing mental health problems and there were also quite a few people with professional health care positions who sought to receive additional training. Problems also varied in character from the more serious (schizophrenia) to somewhat less serious (panic attacks) and from the chronic to the episodic.

My son has for many years experienced depression and more recently possibly some kind of psychosis. He admitted himself into a mental health hospital and received help from psychologists on release. He is currently taking anti-depression medication, but I was not sure he would continue to take the drugs.

I was with a friend who had an anxiety attack which included difficulty in breathing, racing heart, chest pains, cold clammy skin and obvious distress.

As I am a trained nurse and work in a Day Room. We have quite a lot of patients with problems. I found the course very informative as my training 40 years ago lacked in psychiatry. My knowledge comes from life and reading and nowadays I can recognise panic attacks, depression and look after these much better.

The large majority of respondents felt they were able to be helpful with the situation faced. Those who could not help were asked "What was the reason(s) that you were not able to help that person(s)?" The three responses to this question referred to the fact that the person was already getting help or did not want help.

#### Can you give us any examples of something you did?

There was a wide variety of response here. While each response is unique, several broad themes emerged. The foremost theme, especially common for problems with family and friends, was calming the distressed person and listening to them. A sub-theme concerned listening and also actively referring for more specialist assistance.

I helped calm the person, kept them safe with supportive doctor and family members. I also supported without agreeing with their delusions and thoughts.

Listened. Identified that I could understand somewhat of how they saw things. Encouraged to consult a doctor or psychologist.

Two other major themes ran a roughly equal second place. One concerned referral to specialist help, which was more obvious among respondents where the contact was through a professional, workplace situation.

Persuade the person to seek counselling. Tried to listen and advise, also gave essential financial assistance.

The other main theme concerned giving concrete support and practical help and information/advice.

Offered non-discriminatory, non-judgmental support. Made gentle suggestions about ways she might get child to school, helped her get a Christmas tree up (for the kids) which alleviated her guilt around not feeling like having a Christmas.

#### What do you think were the effects on that person/s of what you did?

A few people responded to this question with general, positive statements such as "helpful" or "excellent" effects. The largest group, however, described immediate impacts, which involved calming, comforting or emotional support.

A calm and relaxed atmosphere brought the client a reassurance that I was not a threat to him, so calmed the client down so he could talk and get what was in his mind out and not be prejudged.

The second largest group overall described long term changes. These included getting the person on medication, giving practical help and establishing better relationships.

Kept him alive during the first severe stages of his illness (about one month), reassurance, gave him the time and space to recover himself, cut to a small degree the despair of "utter aloneness", did a lot of simple things (phone calls, tax, Centrelink, bills, forms etc) that for the duration of the illness were impossible for him but helped keep his "life on track".

It cannot be ascertained for certain, but one sensible interpretation of the contrast between these two groups is that more focus was placed on the immediate effects when the respondent was less heavily involved with the person experiencing the mental health problem and/or the problem was less severe. Where the problem was more severe, and especially when the respondent was more closely involved (e.g. friend or family member) more detailed answers, with a longer time frame, were supplied.

#### Can you give us any examples of how your relations with that person/s, or your feelings towards them, have changed?

The overwhelming trend in the data here was in the direction of very positive answers, with a relative trickle of answers that were either mixed or negative. Within the positive trend, two sub-trends could be identified, reflecting the double- barrelled character of the question. In the first, the respondent concentrated mainly upon how the relationship improved:

Our relationship has improved. Communication is better and violent mood swings are not as frequent.

The second theme, of course, focused more on positive improvement in the way the respondent felt or thought.

More tolerant – I'm less inclined to get "cross" about my time being "wasted".

A few respondents gave a negative response, although none of these suggested that the course was in any way deficient or that the things they learned failed to work.

Not a lot. Perhaps they are more willing to communicate their feelings.

Clearly, the experience of the course had positive effects for the large majority of people, either in improving their relationship with someone experiencing a mental health problem or in their feelings towards and ideas about that person, or both.

Importantly, here and elsewhere in the analysis, no hint emerged that the course led people into an unrealistic position. For example, no one told a story that suggested they became over-confident and hence made a mess of a situation.

#### Do you think this change had any effect on the person/s, either good or bad?

The overwhelming response here was that effects had been positive. In a number of cases the respondent simply said "good", or "positive", but many others elaborated.

I believe the change has had a good effect, she is gradually moving forward to make things better in her life with the support of services, GP, etc.

This change has been very positive on both of us. I have seen first hand the benefits of being a good listener and friend and giving helpful advice. My friend states that she is more patient and understanding of others.

Very few overall gave negative responses. Clearly, the dominant response to this question was that the knowledge and skills assisted people to take a constructive and positive response which led to desirable and welcome outcomes.

#### How (if at all) has doing the MHFA course changed how you relate to or feel about the person(s) suffering from that mental health problem?

Two main themes emerged in the answers here – competence and empathy – both with sub-themes. The largest single group of answers concerned professional competence. A substantial number of people who did the course worked with people in the broad area of health or the helping professions. For these respondents, the course clearly delivered a greatly increased sense of comfort and confidence.

It has opened my eyes to the wide range of mental health problems people may suffer. In my work I deal with unemployed people who may be suffering from mental health problem as a result of unemployment or it may be affecting their ability to hold down employment.

While professional competence was important, a linked but smaller theme was personal competence, that is, the capacity to deal confidently with something that had previously been an on-going challenge.

The course provided me the gateway to education about mental health issues, it has made me more self aware and prepared me better for the experiences that I have had thus far especially as it was emotionally hard for me to accept that a close family member was ill.

The second large theme in response to this question centred upon having developed empathy and understanding. As with competence, this theme sub-divided, in this case almost equally, into two themes: generalised empathy towards people with a mental illness and specific empathy towards someone with whom there had previously been tensions and strain.

I have deeper understanding on what these people actually going through. I have become more empathetic towards them.

Made me much more aware of his problem and to question how much of his earlier behaviour (he is now 48) may have been a result of his mental condition.

A third and minor theme was that the course refreshed or reinforced existing knowledge, skills and attitudes.

It was really a "filling in" and affirmation, and "refresher" of all I had learned and experienced in the past when working with people with cerebral palsy, head injuries, and autism. I learned a little more about other mental health problems.

Again, the responses showed that people did the course with a variety of expectations and needs, yet despite this variety, it succeeded across the board in meeting those varied needs and expectations.

#### Is there anything else you would like to say about the MHFA course and its value for you?

This question generated a particularly rich vein of data. The central trend that informed the vast bulk of comments was an extremely positive view of the program. Indeed, only one or two negative comments were received of any kind. Nonetheless, some themes could be discerned. First, within those who were directly positive, some were rather non-specific, while others gave detailed examples. These included gaining knowledge of how to help, greater confidence in providing help, breaking down barriers to dealing with mental health problems, and making contact with other people with similar concerns about mental health issues.

This was some of the best information I've had for years and wish I had done a course this good 30 years ago.

I found the course quite confronting. The insight gained on my personal situation was appreciated. The value of the course to me was high. I would encourage everyone to attend. I think the handbook is an excellent tool. I learnt a lot from the course.

Extremely valuable from point of view of year Advisor at a large rural high school. Provided information and avenues where to get help or refer young people to. Also valuable as a legal studies teacher (an added benefit).

Another group were very enthusiastic about the course and wanted to see it extended, either by follow on courses or by linking to new audiences.

I think it should run regularly as the population needs to learn how to deal with the situations and it creates an awareness and perhaps takes away the fear of the unknown.

This course was exceptional in its presentation and content. A more in-depth and extensive follow up program would be beneficial and valuable.

Finally, another group offered criticism. This was almost always constructive and linked to positive evaluation.

I found it very interesting, but would have liked a little more in-depth treatment of some conditions, with some various "case histories." That would have been very helpful. There seems to be a bit of a gap between how community and mental health departments are portrayed, and what they actually deliver, or take responsibility for.

## Discussion

The data strongly indicate the positive value of the MHFA course. It is true that this was a non-random, availability sample and that such samples can always be challenged on the basis that they lack representation and reflect the views only of a self-selected sample, in this case perhaps of the more satisfied clients. However, if there really were some dissatisfied clients in the population, it seems very likely that at least a few would have wanted to put a critical point of view, but none were found in this study.

Moreover, the findings of this study fit with earlier quantitative evaluations which showed that the course was well received and this allows one to be even more confident of the utility of the data [10,14,15].

The qualitative data allow some more specific points to emerge beyond basic satisfaction and at least five key points should be made. First, the majority of respondents had had some direct experience, post-course, of a situation where mental health issues were salient and it is clear that for this group the course enabled them to take steps that led to better effects than otherwise might have been the case.

Second, it is clear that positive effects were experienced both intra-personally (increased empathy and understanding, increased confidence to act appropriately) as well as inter-personally (increased capacity to handle crises, manage strained relationships, offer help in an effective way, etc.)

Third, and perhaps most strikingly, the positive effects were experienced by a wide range of people including: professionals attending the course to extend their competence; family members/friends attending the course to deal with a pressing problem in their network; and individuals who themselves had experienced mental health problems. These and other groups had varied expectations and needs, yet the course was able to meet the needs of all of these groups with high levels of effectiveness.

Fourth, there is an important absence of certain kinds of potentially bad news. Any well-received course dealing with a sensitive topic runs the risk of being almost too successful. The danger would be that, flushed with enthusiasm and over-confidence, neophytes may rush in where angels fear to tread, not appreciating the dangers of their actions. If the MHFA course has had that effect, there is certainly not a trace of it in these data. This is an important and positive absence. A possible reservation on this conclusion is that those who were over-confident might not have necessarily recognized that this was the case.

Finally, those who attended were able to identify quite specific benefits and many thought the course not only immensely useful, but were keen to see it repeated and extended.

## Conclusion

Examination of the post-course experiences of participants confirms the success of the MHFA course in encouraging supportive actions towards people with mental health problems. These results confirm the earlier quantitative findings from controlled trials.

## Competing interests

BAK and AFJ were developers of the Mental Health First Aid course.

## Authors' contributions

AFJ secured funding, was involved in the design of the study and co-wrote the paper.

BAK was involved in the design of the study and its management.

SKM had a major role in devising the questionnaire, analyzed the responses and co-wrote the paper.

All authors read and approved the final manuscript.

## Pre-publication history

The pre-publication history for this paper can be accessed here:


